# Supplementary material for: Magnitude and determinants of drug-related problems among patients admitted to medical wards of southwestern Ethiopian hospitals: A multicenter prospective observational study
Source: PLoS One. 2021 Mar 16;16(3):e0248575. doi: 10.1371/journal.pone.0248575 (PMC7963049; doi:10.1371/journal.pone.0248575)
Supplement: Attachment — Submitted filename: PONE-D-20-30167_reviewer.pdf [file pone.0248575.s001.pdf]

PLOS ONE

Magnitude and Determinants of Drug Related Problems among Patients Admitted to Medical wards of Ilu Ababor and Buno bedele zonal Hospital: A Multicenter Prospective Observational Study

--Manuscript Draft--

**Manuscript Number:**	PONE-D-20-30167
**Article Type:**	Research Article
**Full Title:**	Magnitude and Determinants of Drug Related Problems among Patients Admitted to Medical wards of Ilu Ababor and Buno bedele zonal Hospital: A Multicenter Prospective Observational Study
**Short Title:**	Magnitude and Determinants of Drug Related Problems among Patients Admitted to Medical wards
**Corresponding Author:**	Firomsa Bekele, Msc<br>Mettu university<br>Mettu, Oromia ETHIOPIA
**Keywords:**	Drug related problem, hospitalized patients, medical ward, Ethiopia
**Abstract:**	Background: Drug related a probl[illegible]s ‘an event or circumstance involving drug therapy that actually or potentially interferes with desired health outcomes. Unrecognized and/or unresolved DRPs can potentially lead to significant drug-related morbidity and/or mortality. Despite this, there were scanty of studies regarding drug therapy problems among patients admitted to medical ward in Ethiopia. Therefore, this study aimed to assess the magnitude of drug-related problems and associated factors among patients admitted to the medical wards of Mettu karl, Bedele and Darimu hospital.<br>Patients and methods: A multicenter prospective cross-sectional study was conducted on patients admitted to the medical ward. Identified DRPs were recorded and classified using the Pharmaceuti[illegible] care network Europe foundation classification system and adverse drug reaction was assessed by using the Naranjo algorithm of adverse drug reaction probability scale. Multivariabe logistic regression was used to analyze the associations between dependent variable and independent variables.<br>Result: Of the 313 study participants, a total of 178 (56.9%) were male. The prevalence of actual or potential DT[illegible]s among subjects put on at least a single drug was fo[illegible] to be 212(67.7%). A total of 331 DRPs were identified on average, 1.057 DRPs p[illegible] patient. The three-leading category of drug-related problems found to be a culprit among the sample were unnecessary 92(27.79%), non-adherence 57(17.22%) and dose too high which a[illegible]unts 56(16.92%). The most common agents associated with drug therapy problem[illegible]ere ceftriaxone 61(28.37%), cimetidine 32(14.88%), and diclofenac 31(14.42%). Area of residence (AOR=2.550,95CI%:1.238-5.253,P=0.011), Length of hospital stay (AOR=9.785,95CI%: 4.668-20.511,P=<0.001), poly pharmacy(AOR=3.229,95CI%: 1.433-7.278, P=0.005) were found to be a predictor drug related problem in multivariable logistic regression analysis.<br>Conclusion: The magnitude of drug therapy problems in patients admitted to the medical ward of Mettu karl hospital, Bedele hospital, and Darimu hospital were found to be high. Therefore, the clinical pharmacy service should be established in hospitals to tackle any drug-related problems in our study area and health care providers of the hospitals need to create awareness of the patients seeking care from health facility about the importance of rational drug use.
**Order of Authors:**	Firomsa Bekele, Msc
	Tesfaye Tsegaye
	Efrem Negash
	Ginenus Fekadu
**Additional Information:**	
**Question**	**Response**

Powered by Editorial Manager® and ProduXion Manager® from Aries Systems Corporation

**Financial Disclosure**<br><br>Enter a financial disclosure statement that describes the sources of funding for the work included in this submission. Review the submission guidelines for detailed requirements. View published research articles from *PLOS ONE* for specific examples.<br><br>This statement is required for submission and **will appear in the published article** if the submission is accepted. Please make sure it is accurate.<br><br>**Unfunded studies**<br>Enter: *The author(s) received no specific funding for this work.*<br><br>**Funded studies**<br>Enter a statement with the following details:<br>• Initials of the authors who received each award<br>• Grant numbers awarded to each author<br>• The full name of each funder<br>• URL of each funder website<br>• Did the sponsors or funders play any role in the study design, data collection and analysis, decision to publish, or preparation of the manuscript?<br>• **NO** - Include this sentence at the end of your statement: *The funders had no role in study design, data collection and analysis, decision to publish, or preparation of the manuscript.*<br>• **YES** - Specify the role(s) played.<br><br>* typeset	The author(s) received no specific funding for this work.
**Competing Interests**<br><br>Use the instructions below to enter a competing interest statement for this submission. On behalf of all authors, disclose any competing interests that could be perceived to bias this work—acknowledging all financial support and any other relevant financial or non-financial competing interests.<br><br>This statement **will appear in the published article** if the submission is accepted. Please make sure it is	The authors have declared that no competing interests exist.

accurate. View published research articles from *PLOS ONE* for specific examples.<br><br>**NO authors have competing interests**<br><br>Enter: *The authors have declared that no competing interests exist.*<br><br>**Authors with competing interests**<br><br>Enter competing interest details beginning with this statement:<br><br>*I have read the journal's policy and the authors of this manuscript have the following competing interests: [insert competing interests here]*<br><br>* typeset	
**Ethics Statement**<br><br>Enter an ethics statement for this submission. This statement is required if the study involved:<br><br>• Human participants<br>• Human specimens or tissue<br>• Vertebrate animals or cephalopods<br>• Vertebrate embryos or tissues<br>• Field research<br><br>Write "N/A" if the submission does not require an ethics statement.<br><br>General guidance is provided below. Consult the submission guidelines for detailed instructions. **Make sure that all information entered here is included in the Methods section of the manuscript.**	Ethical clearance was obtained from the ethics review board of Mettu University. Permission was obtained from medical director of the MKH, DH and BH to access medical ward patients and conducts the study. The benefit and risks of the study was explained to each participant included in the study and oral informed consent was be obtained from each patient involved in the study. To ensure confidentiality, name and other identifiers of patients and health care professionals was not recorded on the data collection tools.

**Format for specific study types**<br><br>**Human Subject Research (involving human participants and/or tissue)**<br>• Give the name of the institutional review board or ethics committee that approved the study<br>• Include the approval number and/or a statement indicating approval of this research<br>• Indicate the form of consent obtained (written/oral) or the reason that consent was not obtained (e.g. the data were analyzed anonymously)<br><br>**Animal Research (involving vertebrate animals, embryos or tissues)**<br>• Provide the name of the Institutional Animal Care and Use Committee (IACUC) or other relevant ethics board that reviewed the study protocol, and indicate whether they approved this research or granted a formal waiver of ethical approval<br>• Include an approval number if one was obtained<br>• If the study involved *non-human primates*, add *additional details* about animal welfare and steps taken to ameliorate suffering<br>• If anesthesia, euthanasia, or any kind of animal sacrifice is part of the study, include briefly which substances and/or methods were applied<br><br>**Field Research**<br><br>Include the following details if this study involves the collection of plant, animal, or other materials from a natural setting:<br>• Field permit number<br>• Name of the institution or relevant body that granted permission	
**Data Availability**<br><br>Authors are required to make all data underlying the findings described fully available, without restriction, and from the time of publication. PLOS allows rare exceptions to address legal and ethical concerns. See the PLOS Data Policy and FAQ for detailed information.	Yes - all data are fully available without restriction

Powered by Editorial Manager® and ProduXion Manager® from Aries Systems Corporation

A Data Availability Statement describing where the data can be found is required at submission. Your answers to this question constitute the Data Availability Statement and **will be published in the article**, if accepted.<br><br>**Important:** Stating 'data available on request from the author' is not sufficient. If your data are only available upon request, select 'No' for the first question and explain your exceptional situation in the text box.<br><br>Do the authors confirm that all data underlying the findings described in their manuscript are fully available without restriction?	
**Describe where the data may be found in full sentences. If you are copying our sample text, replace any instances of XXX with the appropriate details.**<br><br>• If the data are **held or will be held in a public repository**, include URLs, accession numbers or DOIs. If this information will only be available after acceptance, indicate this by ticking the box below. For example: *All XXX files are available from the XXX database (accession number(s) XXX, XXX.).*<br>• If the data are all contained **within the manuscript and/or Supporting Information files**, enter the following: *All relevant data are within the manuscript and its Supporting Information files.*<br>• If neither of these applies but you are able to provide **details of access elsewhere**, with or without limitations, please do so. For example:<br><br>*Data cannot be shared publicly because of [XXX]. Data are available from the XXX Institutional Data Access / Ethics Committee (contact via XXX) for researchers who meet the criteria for access to confidential data.*<br><br>*The data underlying the results presented in the study are available from (include the name of the third party*	Available only after acceptance

and contact information or URL).<br>• This text is appropriate if the data are owned by a third party and authors do not have permission to share the data.<br><br>* typeset	
Additional data availability information:	Tick here if the URLs/accession numbers/DOIs will be available only after acceptance of the manuscript for publication so that we can ensure their inclusion before publication.

Powered by Editorial Manager® and ProduXion Manager® from Aries Systems Corporation

Manuscript

Click here to access/download;Manuscript;DTP METTU MANUSCRIPT FINAL.docx

Magnitude and Determinants of Drug Related Problems among Patients Admitted to Medical wards of Ilu Ababor and Buno bedele zonal Hospital: A Multicenter Prospective Observational Study

Firomsa Bekele[1*], Tesfaye Tsegaye[1], Efrem Negash[2], Ginenus Fekadu[3],

[1]Department of Pharmacy, College of Health Science, Mettu University, Mettu, Ethiopia
[2]Department of Public health, College of Health Science, Mettu University, Mettu, Ethiopia
[3]Department of Pharmacy, Institute of Health Science, Wollega University, Nekemte, Ethiopia

***Corresponding author:**
Firomsa Bekele
Department of pharmacy, College of health science
Mettu University, Mettu, Ethiopia
E-mail: firomsabekele21@gmail.com

Abstract

Background: Drug related a problem is ‘an event or circumstance involving drug therapy that actually or potentially interferes with desired health outcomes. Unrecognized and/or unresolved DRPs can potentially lead to significant drug-related morbidity and/or mortality. Despite this, there were scanty of studies regarding drug therapy problems among patients admitted to medical ward in Ethiopia. Therefore, this study aimed to assess the magnitude of drug-related problems and associated factors among patients admitted to the medical wards of Mettu karl, Bedele and Darimu hospital.

Patients and methods: A multicenter prospective cross-sectional study was conducted on patients admitted to the medical ward. Identified DRPs were recorded and classified using the Pharmaceutical care network Europe foundation classification system and adverse drug reaction was assessed by using the Naranjo algorithm of adverse drug reaction probability scale. Multivariabe logistic regression was used to analyze the associations between dependent variable and independent variables.

Result: Of the 313 study participants, a total of 178 (56.9%) were male. The prevalence of actual or potential DTPs among subjects put on at least a single drug was found to be 212(67.7%). A total of 331 DRPs were identified on average, 1.057 DRPs per patient. The three-leading category of drug-related problems found to be a culprit among the sample were unnecessary 92(27.79%), non-adherence 57(17.22%) and dose too high which accounts 56(16.92%). The

most common agents associated with drug therapy problems were ceftriaxone 61(28.37%), cimetidine 32(14.88%), and diclofenac 31(14.42%). Area of residence (AOR=2.550,95CI%:1.238-5.253,P=0.011), Length of hospital stay (AOR=9.785,95CI%: 4.668-20.511,P=<0.001), poly pharmacy(AOR=3.229,95CI%: 1.433-7.278, P=0.005) were found to be a predictor drug related problem in multivariable logistic regression analysis.

Conclusion: The magnitude of drug therapy problems in patients admitted to the medical ward of Mettu karl hospital, Bedele hospital, and Darimu hospital were found to be high. Therefore, the clinical pharmacy service should be established in hospitals to tackle any drug-related problems in our study area and health care providers of the hospitals need to create awareness of the patients seeking care from health facility about the importance of rational drug use.

Keywords: Drug related problem, hospitalized patients, medical ward, Ethiopia

Background

Drug related problems is ‘an event or circumstance involving drug therapy that actually or potentially interferes with desired health outcomes ‘and it is classified as dosing problems, adverse drug reaction, drug- drug interaction, inappropriate prescription and patient adherence to the drug [1,2]. Although medications play a vital role in the cure, palliation, and inhibition of disease, they also expose patients to drug-related problems (DRPs) [3].

DRPs may arise at all stages of the medication process from prescription to follow-up of treatment. Most of the problem usually occurs on administration, dispensing and during the patient’s use of a medicinal product, but the lack of proper follow-up and reassessment of medical treatment by the physicians also a major problem[4].

Due to several reasons, hospitalized patients are likely to receive more medications than those treated as outpatients [5]. It is estimated that approximately 5 to 10% of all hospital admissions are drug related, and about 22% of patients are discharged with DTPs. As many as 28% of all emergency department visits are drug related. Common drug therapy problems resulting in emergency department visits are adverse drug reactions, noncompliance, and inappropriate prescribing [6].

DRPs are a major safety issue for hospitalized patients and it may lead to reduced quality of life, increased hospital stay, increased overall health care cost and even increases risk of morbidity and mortality [7]. More people die of inappropriate drug treatment than from breast cancer, AIDS and traffic accidents all together [8].

The economic burden arising from drug related morbidity and mortality in United State of America (USA) was $177.4 billion annually[1] Whereas, £100707 was reported in Australian study(9). A study done in Lagos State University Teaching Hospital, Nigeria, reported that approximately1.83 million naira (15,466.60 USD) was spent on managing all the patients admitted due to ADRs. Therefore, DRPs are major area concern of the patient's economic burden to the patients as well as to the whole society. Hence, improving drug therapy by preventing drug-related problems may have an important effect on the patients treatment related cost [3,7].

Adverse drug reactions may cause patients to lose confidence in or have negative emotions toward their physicians and seek self-treatment options, which may consequently precipitate additional ADRs. Around 5% of all hospital admissions are the result of an ADR, and around 10%–20% of inpatients will have at least one ADR during their hospital stay [10].

The study conducted in Australian showed that about 1.3 million People are injured each year due to medication errors and ADEs occurred in 16.6% of admissions, resulting in permanent disability in 13.7% of patients and death in 4.9% [11].

Hospitalized patients are more likely exposed to poly pharmacy. This in turn is a concern for potential drug related problems. Some of the DRPs exist at the time of admission to hospital, while others arise during hospital management. On average 2.6 DRPs occur per patient in internal medicine wards in Norway and the presence of DRPs increased approximately linearly with the number of drugs used [12].

Studies worldwide indicate that despite the availability of effective medical therapy, more than half of patient's treatment was poorly controlled. This is mostly because of drug therapy problems which include but not limited to non-compliance, ADRs, improper drug selection and drug interactions [13].

Therefore, Several studies conducted in different hospital showed a high incidence of drug related problems among hospitalized patients. However, there was no studies conducted related to drug therapy problems in medical wards of Ilubabor and Buno bedele zone and so this study was aimed to assess drug related problem and its predictors in patients with medical ward of Mettu karl, Bedele, and Darimu hospital.

Patients and methods

Study setting, design and study period

A multicenter prospective observational study was conducted at in three hospital found in the Ilu ababor and Buno bedele zone namely Mettu,Bedele and Darimu hospital from March 1, 2020 to June 1, 2020. Iluababor and Buno bedele zone are found in southwest Ethiopia which are 572km and 423km to the southwest of Addis Ababa, the capital city of Ethiopia.

Study participants and eligibility criteria

Patients ≥18 years who were admitted to the non-intensive care unit (ICU) of medical wards and with more than 48 hours of length of stay were included. Patients who refused to participate, re-admitted during the study period, and developed ADR due to genetic factors were excluded.

Study variables and outcome endpoints

The Drug therapy problem was the primary outcome. ADR was assessed by using the Naranjo algorithm of the ADR probability scale [14]. Hill-Bone Compliance to High Blood Pressure Therapy Scale was used to measure medication adherence. For this study purpose, we 9-item medication-taking sub-scale was selected. Each item is a four-point Likert type scale (none of the time, some of the time, most of the time and all of the time) [15, 16]. The total scores on this subscale range from 9 to36 with higher scores reflecting poorer adherence to drug therapy. The median split was used and dichotomized into two groups 1 Adherent to the treatment and 0 =Non-adherent to the medication.

Sample size and sampling technique

Single population proportion formula was used to calculate the required sample size by considering the following assumptions: Proportion of drug related problem is 75.51%[6], 95% confidence level, and 5% margin of error (absolute level of precision).

$$n = \frac{(Z\alpha/2)^2\, p\,(1-p)}{d^2}$$

z= 1.96
P= 75.51% (0.7551)
d= 0.05

$$n = \frac{(1.96)^2\,(0.755)\,(0.245)}{(0.05)^2} = 284.24 \sim 284$$

Where;

n = sample size

A 10% contingency yielded a final sample size of 313. The study subjects were selected by proportional allocation based on the number of the patients that the respective hospitals contain in their medical ward. Accordingly from MKH190, BH 68 and DH55 patients were included. The subjects were chosen by using consecutive sampling technique.

Data collection process and management

Data was collected using questionnaire which was developed after reviewing different literature and supplementary information and clarifications on some patient's medical information were obtained through discussion with the patient and the physician. Four medical doctor, four nurses and four pharmacists were recruited for data collection; three medical doctor and three pharmacist were assigned to supervise the data collection process. The supervisor and principal investigator were closely follow the data collection process at the spot**.** With the data collected, the principal investigator evaluated appropriateness of medical therapy using various references like Medscape, Up-to-date, lexicom and Micromedex and different guidelines. Identified DRPs were recorded and classified using DRP registration format [2]. ADR was assessed by using Naranjo algorithm of ADR probability scale [14]. Five percent of the sample was pre-tested to

check the acceptability and consistency of the data collection tool two weeks before the actual data collection.

Data processing and analysis

The data was entered in to computer using EPI-info 3.5.4 software. Data checking and cleaning was done by principal investigator on daily basis during collection before actual analysis. Analysis was done using statistical software for social sciences (SPSS) 23.0. Descriptive data was generated and placed in terms of frequency and percentage. Results were expressed as proportions and as means ± Standard Deviations (SD). Bivariate and multivariable logistic regression was used to analyze the associations between dependent variable and independent variables. A p-value of less than 0.05 is considered statistically significant.

Ethics approval and consent-to-participate

Ethical clearance was obtained from the ethics review board of Mettu University. Permission was obtained from medical director of the MKH, DH and BH to access medical ward patients and conducts the study. The benefit and risks of the study was explained to each participant included in the study and oral informed consent was be obtained from each patient involved in the study. To ensure confidentiality, name and other identifiers of patients and health care professionals was not recorded on the data collection tools.

Operational definitions

Drug-related problem: In this study it include ADR, non-adherence, inappropriate indication and dose, drug interaction and ineffective drug therapy

Hospital stay: The time gap that was spent by the patient in the hospital from his/her admission till his/her discharge (discharge date was determined by looking his/ her discharge date from his/her medical chart).

Medication error-: Any preventable event that may cause or lead to inappropriate medication use or patient harm while the medication is in the control of the health care professional, patient or consumer.

Pharmaceutical care: The process through which a pharmacist cooperates with a patient and other professionals in designing, implementing and monitoring a therapeutic plan that will produce specific therapeutic outcomes for the patient.

Poly-pharmacy: The daily consumption of 5 or more medications [7].

Co-morbidity: The presence of two or more diseases [17].

Results

Socio-demographic characteristics of study participants

Over the study period, a total of 313 patients were admitted to medical wards of MKH,BH,and DH. A total of 178 (56.9%) were male and almost half 163 (52.1%) of the patients were in the age range of 18-35 years. A total of 171(54.6%) were married and 179(57.2%) were farmer (table 1).

Table 1: Socio-demographic characteristics of the study respondents (N=313)

**Variables**		**Frequency(n)**	**Percent (%)**
Sex	Male	178	56.9
	Female	135	43.1
Age (years)	18-35	163	52.1
	36-64	124	39.6
	≥ 65	26	8.3
Marital status	Married	171	54.6
	Single	95	30.4
	Divorced	31	9.9
	Widowed	16	5.1
Educational status	Illiterate	61	19.5
	Grade 1-8	114	36.4
	Grade 9-12	60	19.2
	Diploma	43	13.7
	Degree and above	35	11.2
Occupation	Farmer	179	57.2
	Merchant/trade	18	5.8
	Government employee	53	16.9

	Housewife	18	5.8
	Student	38	12.1
	Others*	7	2.2
Residence	Urban	70	22.4
	Rural	243	77.6

Others*: Daily laborer, non-governmental employee

Life style and clinical characteristics of the study respondents

Over the study period, a total of 125(36.63%) patiens had one or more co-morbid disease and a total of 127(59.4%) patients had a prolonged hospital stay while the average duration of hospital stay was 7.14 ± 4.731 days. Regarding to their life style,62(19.8%)of them were drink alcohol(table 2).

Table 2: **Life style and clinical characteristics of the study respondents (N=313)**

Variables		Frequency(n)	Percentage (%)
Drink alcohol	Yes	62	19.8
	No	251	80.2
Chew a chat	Yes	45	14.4
	No	268	85.6
Smoke a cigarette	Yes	25	8
	No	288	92
Presence of comorbidity	Yes	125	36.63
	No	188	60.1
Length of hospital stay (days)	<7	186	39.9
	≥7	127	59.4
Number of medications per patient	<5	241	77
	≥5	72	23

Incidence and characterization of DTPs

The prevalence of actual or potential DTPs among subjects put on at least a single drug was found to be 212(67.7%). A total of 331 DRPs were identified on average, 1.057 DRPs per patient. The three-leading category of drug-related problems found to be a culprit among the sample were unnecessary 92(27.79%), non-adherence 57(17.22%) and dose too high which accounts 56(16.92%)(Table 3).

Table 3: Types of drug therapy problems of the patients admitted to medical ward of MKRH,BH and DH

**Types of drug therapy problems**	**Frequency (n)**	**Percentage (%)**
Unnecessary drug therapy	92	27.79%
Non-adherence	57	17.22%
Dose too high	56	16.92%
Needs additional drug therapy	50	15.11%
Dose too low	38	11.48%
Ineffective drug therapy	30	9.06%
ADR	8	2.42%

The common drugs involved in DTPs

The most common agents associated with drug therapy problems were ceftriaxone 61(28.37%), cimetidine 32(14.88%), and diclofenac 31(14.42%)(table 4).

Table 4: Common drugs associated with the occurrence of DRPs among patients admitted to the medical ward of MKRH, BH and DH from June to August, 2020(N=215)

**Individual Drugs**	**Frequency (n)**	**Percentage (%)**
Ceftriaxone	61	28.37
Cimetidine	32	14.88
Diclofenac	31	14.42
Furosemide	26	12.09
Doxycycline	24	11.16

Metronidazole	20	9.30
Tramadol	13	6.05
Augumentin	12	5.58
ASA	10	4.65
Spironolactone	9	4.19

Factors associated with of DTPs

The result of the multivariable logistic regression showed that a significant association was observed between the area of residence, length of hospital stay, and poly-pharmacy with the presence of DRPs. Patients living in rural area were 2.5 times more likely to had at least one drug therapy problems than patients who live in rural area(AOR=2.550,95CI%: 1.238-5.253,P=0.011). Patients whose hospital stays greater than or equal to seven days were 9.8 times more likely to have DRPs than patients whose hospital stay less than seven days (AOR=9.785,95CI%: 4.668-20.511,P=<0.001). Lastly, patients who have prescribed 5 or more drugs (polypharmacy) were 3.2 times more likely to have DRPs than patients prescribed with less than 5 drugs (AOR=3.229,95CI%: 1.433-7.278, P=0.005) (table 5).

Table 5: Multivariable logistic regression analysis result of factors associated with DRP among Patients admitted to Medical Wards of MKRH,BH and DH.

Variables	Category	DTPs		COR (95%CI)	P-value	AOR (95%CI)	P-value
		Yes (n=212)	No (n=101)				
Sex	Male	126 (59.43%)	52(51.49 %)	1.38(0.857-2.22) *	0.185	1.54(0.892-2.665)	0.121
	Female	86(40.57%)	49(48.51 %)	1		1	
Area of residence	Urban	47(22.17%)	23(22.77 %)	1	0 .1905	1	0.011
	Rural	165(77.83%)	78(77.23 %)	0.966 (0.548-1.703) *		2.550(1.238-5.253) **	

Presence of comorbidity	Yes	85(40.09%)	40(39.60%)	1.021 (0.629-1.66) *	0.193	1.41(0.775-2.558)	0.261
	No	127(59.91%)	61(60.40%)	1		1	
Poly pharmacy	Yes	63(29.72%)	9(8.91%)	4.322 (2.051-9.106) *	<0.001	3.229 (1.433-7.278) **	0.005
	No	149(70.28%)	92(91.09%)	1		1	
Length of hospital stay (days)	≥7	113(53.30%)	14(13.86%)	7.093 (3.794-13.259) *	<0.001	9.785 (4.668-20.511) **	<0.001
	<7	99(46.70%)	87(86.14%)	1		1	
Drink alcohol	Yes	47(22.17%)	15(14.85%)	1.633(.864-3.088) *	0.131	2.218(0.959-5.128)	0.063
	No	165(77.83%)	86(85.15%)	1		1	
Smoke cigarette	Yes	18(8.49%)	7(6.93%)	1.246(0.503-3.086) *	0.164	0.445(0.139-1.429)	0.174
	No	194(91.51%)	94(93.07%)	1		1	
Chew a chat	Yes	25(11.79%)	20(19.80%)	0.541(0.285-1.030) *	0.062	1.714(0.775-3.793)	0.183
	No	187(88.21%)	81(80.20%)	1		1	

*Shows significant at p-value 0.25, **Shows statistically significant at p-value 0.05.

AOR: Adjusted odds ratio; COR: Crudes odds ratio

Discussion

This study aimed to determine the factors associated with an increased risk of developing one or more DRP among patients admitted to medical wards. The prevalence of DRP in our study patents was 212(67.7%). which was similar to the study done in Gondar (66%)[7], Northern Sweden (66%)[18]. However, the magnitude of the drug therapy problem was lower than the Dessie referral hospital(75.51%)[6], Kenya (93.8%)[19], Tikur Anbesa Specialized hospital (70.4%)[12], and Jimma University specialized hospital (73.5%)[20],and higher than the study conducted in India in which the overall incidence of DRPs was found to be 47.66%[4].

The large variation seen on the magnitude of DRPs across studies might be due to different classification of systems used to classify DRPs and settings in which DRPs were assessed. Despite the difference seen in the magnitude of DRPs, the finding in our study showed the need to make an intervention on the DRPs so that the patients improve the success of treatment outcomes. Furthermore, we recommend future studies to use similar DRPs classification systems to generate comparable evidence.

In the present study Unnecessary drug therapy 92(27.79%), non-adherence 57(17.22%), and dose too high 56(16.92%)were most frequently encountered DRPs and ADR was the least which accounts 8(2.42%). This is consistent with the study of Dilla university referral hospital in which Non-adherence was the most commonly occurred types of DRP 68(29.69%), and ADR was the least type of DRP 7(3.06%)[21],in university of Gondar in which inappropriate dose was the prevalent DRP[7], India in which ADR was the least occurred types of DRP which accounts 2(1.39%[4].
On the contrary, non-adherence was the least common types of drug therapy problem in Jimma University Specialized Hospital[20]. On the other hand, ADR (18.6%) were the most commoly occurred DRP In Adama hospital medical college[13].

In our study, the most common drugs associated with at least one of the drug therapy problems were ceftriaxone 61 (28.37%), cimetidine 32(14.88%), and diclofenac 31(14.42%). Similarly, the study conducted in Dessie referral hospital revealed that ceftriaxone (25.81%) was the most frequent specific drug prone to the drug therapy problems and non-steroidal anti-inflammatory drugs accounts, 9 (4.84%)[6]. The study conducted in India showed that Antibiotics (33.5%),GIT drugs(29.3%) and NSAID(16.08%) were the most commonly involved in drug related problems[4].

On the contrary, the study conducted in the university of Gondar showed that the most common agents associated with drug therapy problems were omeprazole 45 (17.6%), heparin 22 (8.6%), and aspirin 21 (8.2%)[7]. The study conducted in Hong kong revealed that Gentamicin, ranitidine and fluconazole were most frequently associated with DRPs [22]. The variety of drugs involved in drug therapy problems were due to availability, patients and physician preferences, and different treatment guidelines within different countries.

In current study, 331 DRPs were recorded which is about 1.057 DRPs per patient. This finding is lower than study conducted among Hiwot fana specialized university hospital [23], Kenya [19],but comparable with the study of University of Gondar Teaching Hospital, Northwest Ethiopia[7].The large variation seen on magnitude of DRPs across studies might be due to different classification of system used to classify DRPs, settings in which DRPs were assessed, and difference in sample size of the study participants.

In the present study, we found the association between the risk of DRP and area of the residence. Patients who live in rural area were 2.55 times more likely to develop drug-related problems as compared to patients who were in urban area. This was inconsistent to the study of spain in which Among demographic variables, only female sex was associated with a higher risk of developing at least one DRP[24]. Similarly the area of residence was not a determinant of DRP in JUSH [25]. The high risk of DRP in rural area was due to lack of access to health information as the result of long distance to reach the health facilities.

The logistic regression analysis showed that the number of drugs prescribed per patients (≥5 prescribed drugs) was strong predictors for DRP occurrence. Patients who took ≥5 drugs were 3.23 times more likely to develop drug-related problems as compared to patients who took < 5 drugs. This was consistent to the study of United Kingdom and Saudi Arabia[26], Hong kong[22], Singapore[27],Dilla University Referral Hospital[21], and Jimma University Specialized Hospital[25]. This might be due to the potential drug drug interaction as the results of poly-pharmacy

In our study, patients who had prolonged hospital stay were highly risk for the developments of at least one types of DTP. Patients whose hospital stay was ≥7 days, were 9.8 times more likely to have DRPs than patients whose hospital stay <7days**.** This was consistent with that of university of Gondar [7]. On the contrary, in Jimma's university specialized hospital the length of hospital stay didn't predict the presence of drug-related problems [20]. This is due to the

patients who had prolonged hospital stay may develop different nosocomial infections that need complex therapeutic management.

As the limitations, the study was cross-sectional study which is difficult to determine the causal effect relationship**.** In addition, we only evaluate drug therapy problems among patients admitted to the medical ward which lacks generalizability. As the strength the study was multicenter and prospective as well as information on different organ function tests (renal, liver), diagnostic and laboratory tests were used to assess any drug therapy problems.

Conclusion

The magnitude of drug therapy problems in patients admitted to the medical ward of MKH, BH, and DH were found to be high. Unnecessary drug therapy and ADR were the most and the least prevalent DRPs respectively. Ceftriaxone, cimetidine, diclofenac, furosemide, doxycycline, Metronidazole, tramadol, Augmentin, ASA, and spironolactone were the most common drugs involved in drug related problems. Area of the residence, poly-pharmacy, and prolonged hospital stay were determinants of drug therapy problems. Therefore, the clinical pharmacy service should be established in hospitals to tackle any drug-related problems in our study area and health care providers of the hospitals need to create awareness of the patients seeking care from health facility about the importance of rational drug use.

Abbreviations

ADE: Adverse Drug Event; ADR: Adverse drug reaction; AIDS: AIDS: Acquired immune deficiency disease; ASA: Acetyl Salicylic Acid; AOR: Adjusted odds ratio; BH: Bedele Hospital; COR: crude odds ratio; DH: Darimu Hospital: DRP: Drug related problem; DTP: Drug therapy problem; GIT: Gastro Intestinal Drug; ICU: Intensive care unit; MKH: Mettu Karl Hospital; NSAID: Non-Steroidal Inflammatory Drug; SD: Standard Deviation; SPSS: statistical package for social sciences; USA: United States of America; USD: United State Dollar

Acknowledgment

We thank Mettu University for logistic support. We are grateful to staff members and health care professionals of Metu karl, Bedele, and Darimu hospital, data collectors, and study participants for their cooperation in the success of this study.

Authors' Contributions

FB and TT contributes in the proposal preparation, study design, analysis and writes up the manuscript. EN and GF contributed to the design of the study, and writes up the manuscript. All authors read and approved the final version of the manuscript.

Consent for publication: Not applicable. No individual person's personal details, images, or videos are being used in this study.

Funding: None

Availability of data and materials

The datasets used and/or analyzed during the current study are available from the corresponding author on reasonable request.

Competing interests: No competing interests exist.

References

1. Pharmaceutical Care Network Europe Foundation 2010. Classification for drug related problems revised V 6.20. page 2.

2 . Cipolle R, Strand L, Morley P, 2004, Pharmaceutical care practice: the clinicians guide, New York: McGraw Hill.

3.Mequanent Kassa Birarra,Tigist Bacha Heye and Workineh Shibeshi Assessment of drug-related problems in pediatric ward of Zewditu Memorial Referral Hospital, Addis Ababa, Ethiopia. Int J Clin Pharm DOI 10.1007/s11096-017-0504-9

4. Sarfaraz Mohammed, Sanjay Poudel, Fernance Laloo, Arjun Madhur, Rinson Robert, Binu Mathew. Assessment of drug related problems in Tertiary care teaching hospital, India. *Asian J Pharm Clin Res, Vol 10, Issue 2, 2017, 310-313*

5.. Flaherty JH, Perry HM III, Lynchard GS, Morley JE. Polypharmacy and hospitalization among older home care patients. J Gerontol A Biol Sci Med Sci. 2000;55(10):M554–9.

6. Yaschilal Muche Belayneh, Gedefaye Amberbir and Asrat Agalu Aprospective observational study of drug therapy problems in medical ward of a Dessie referral hospital in northeast

Ethiopia. BMC Health Services Research (2018) 18:808. https://doi.org/10.1186/s12913-018-3612-x
7. Mekonnen GB, Birarra MK, Tekle MT, Bhagavathula AS. Assessment of Drug Related Problems and its Associated Factors among Medical Ward Patients in University of Gondar Teaching Hospital, Northwest Ethiopia: A Prospective Cross-Sectional Study. J Basic Clin Pharma 2017;8:S016-S021.
8.Kohl L, Corrigan J and Donaldson M (Eds) committee on quality of health care in America, institute of medicine, 1999, to err is human: building a safer health system, Washington, DC: National Acadamy Press
9. Easton KL, Chapman CB, Brien JA. Frequency and characteristics of hospital admissions associated with drug-related problems in paediatrics. Br J Clin Pharmacol 2004;57:611-15.
10.Stephanie N. Schatz and Robert J. Weber. Adverse Drug Reactions. PSAP 2015 • CNS/Pharmacy Practice
11.Bosma L, Jansman F, Franken A, Harting J, Vanden B, 2007, Evaluation of Pharmacist clinical intervention in Dutch hospital setting, Pharm World Sci; 30(1): 31-38.
12.Ayalew MB,Megersa TN and Mengistu YT. Drug related problems in medical ward of tikur Anbessa specialized hospital,Ethiopia. J Res pharm pract 2015 oct-Dec;4(4):216-21.doi:10.4103/2279-042x.167048
13. Hussein M, Lenjisa JL, Woldu MA, Tegegne GT, Umeta GT, et al. (2014) Assessment of Drug Related Problems Among Hypertensive Patients on Follow up in Adama Hospital Medical College, East Ethiopia. Clinic Pharmacol Biopharmaceut 3: 122. doi:10.4172/2167-065X.1000122
14.Naranjo CA et al. A method for estimating the probability of adverse drug reactions. Clin Pharmacol Ther 1981; 30: 239245.
15. Song Y, Han HR, Song HJ, Nam S, Nguyen T, Kim MT. Psychometric evaluation of hill-bone medication adherence subscale. Asian Nurs Res. 2011 ;5(3):183– 188. doi:10.1016/j. anr.2011.09.007
16. Kim MT, Hill MN, Bone LR, Levine DM. Development and testing of the hill-bone compliance to high blood pressure therapy scale. Prog Cardiovasc Nurs. 2000 ;15(3):90 – 96. doi:10.1111/j.1751- 7117.2000.tb00211.x

17. Tigabu BM, Daba D, Habte B. Drug-related problems among medical ward patients in Jimma university specialized hospital, Southwest Ethiopia. Journal of research in pharmacy practice. 2014 Jan;3(1):1.

18. Cecilia Peterson and Maria Gustafsson. Characterisation of Drug-Related Problems and Associated Factors at a Clinical Pharmacist Service-Naı¨ve Hospital in Northern Sweden. Drugs - Real World Outcomes (2017) 4:97–107.DOI 10.1007/s40801-017-0108-7

19. Degu A, Njogu P, Weru I, Karimi P. Assessment of drug therapy problems among patients with cervical cancer at Kenyatta National Hospital, Kenya. Gynecologic oncology research and practice. 2017 Dec;4(1):15.

20. Bereket Molla Tigabu, Daniel Daba, Belete Habte, Drug‑related problems among medical ward patients in Jimma university specialized hospital, Southwest EthiopiaJournal of Research in Pharmacy Practice / Jan-Mar 2014 / Vol 3 / Issue 1. DOI: 10.4103/2279-042X.132702

21. Bekele NA, Hirbu JT. Drug Therapy Problems and Predictors Among Patients Admitted to Medical Wards of Dilla University Referral Hospital, South Ethiopia: A Case of Antimicrobials. Infection and Drug Resistance. 2020 Jun 12;13:1743-50.

22.Asia N. Rashed,Lynda Wilton, Charles C. H. Lo,Benjamin Y. S. Kwong,Suzanne Leung& Ian C. K. Wong. Epidemiology and potential risk factors of drug-related problems in Hong Kong paediatricwards.British Journal of ClinicalPharmacology.77:5/873–879. DOI:10.1111/bcp.12270

23. Ayele Y, Melaku K, Dechasa M, Ayalew MB, Horsa BA. Assessment of drug related problems among type 2 diabetes mellitus patients with hypertension in Hiwot Fana Specialized University Hospital, Harar, Eastern Ethiopia. BMC research notes. 2018 Dec;11(1):1-5.

24. Olatz Urbina,Olivia Ferrández,Sònia Luque, Santiago Grau,Sergi Mojal and Rosa Pellicer etal Patient risk factors for developing a drug-related problem in a cardiology ward. Therapeutics and Clinical Risk Management 2015:11 9–15

25. Niriayo YL, Kumela K, Kassa TD, Angamo MT (2018) Drug therapy problems and contributing factors in the management of heart failure patients in Jimma University Specialized Hospital, Southwest Ethiopia. PLoS ONE 13(10): e0206120. https://doi.org/10.1371/journal.pone.0206120

26. Al Hamid A, Aslanpour Z, Aljadhey H, Ghaleb M. Hospitalisation resulting from medicine-related problems in adult patients with cardiovascular diseases and diabetes in the United

Kingdom and Saudi Arabia. International journal of environmental research and public health. 2016 May;13(5):479.

27. Koh Y, Kutty FB, Li SC. Drug-related problems in hospitalized patients on polypharmacy: the influence of age and gender. Therapeutics and clinical risk management. 2005 Mar;1(1):39.
