# Supplementary material for: Magnitude and determinants of drug-related problems among patients admitted to medical wards of southwestern Ethiopian hospitals: A multicenter prospective observational study
Source: PLoS One. 2021 Mar 16;16(3):e0248575. doi: 10.1371/journal.pone.0248575 (PMC7963049; doi:10.1371/journal.pone.0248575)
Supplement: Attachment — Submitted filename: plose one DTP esponse letter.docx [file pone.0248575.s003.docx]

M. Mahmud Khan

Editor, PLOS ONE

Dear Editor in chief of the Manuscript PONE-D-20-30167 entitled " Magnitude and Determinants of Drug Related Problems among Patients Admitted to Medical wards of Ilu Ababor and Buno bedele zonal Hospital: A Multicenter Prospective Observational Study," submitted to PLOS ONE. Thanks for your time and consideration in editing and reviewing the manuscript. We have carefully read your comments and corrected inline of your comments and suggestions. All comments raised were edited and incorporated in the revised manuscript.

Here are the responses and elaborations for the comments from editor and reviewer!

**EDITORS COMMENTS**

***Editor comment:*** The paper should provide more information on drugs used by patient characteristics, i.e., major drugs with problems can be cross-tabulated with age, morbidity status, etc.

*Author response:* In table 5 of revised manuscript most common drugs was cross tabulated with the variables like sex, residence, co-morbidity and age

***Editor comment:*** In the introduction, authors mentioned that a significant proportion of patients get admitted to hospitals due to adverse drug events. What approach did the study follow to ensure that the patient was not admitted due to adverse drug reactions?

*Author response:* ADR was assessed by using the Naranjo algorithm of the ADR probability scale

***Editor comment:*** Define the drug therapy problem "Unnecessary drug therapy" and since it is quite high, is there some specific patient groups that are more likely to be affected by this? Does it indicate error in physician prescription?

*Author response:* In operational definition we have added the definition and criteria of unnecessary drug therapy. Patients presents with infectious disease were highly affected by unnecessary drug therapy because in our study are ceftriaxone was used unnecessarily. Any medication error occurred during prescribing, administration and dispensing was considered as unnecessary drug therapy

***Editor comment:*** Paper needs thorough editing and revisions.

*Author response:* The whole manuscript was edited as per your comments

***Editor comment:*** Methodology should be clearly presented (see the reviewer #1 comment on this as well)

*Author response:* We carefully revised and edited the methodology part

***Editor comment:*** Regarding o Journal Requirements

*Author response:* The manuscript was written as per PLOS ONE's style

***Editor comment:*** Please amend your current ethics statement to include the full name of the ethics committee/institutional review board(s) that approved your specific study.

*Author response:* The full name of the ethics committee/institutional review board(s) was written in revised manuscript and Ethics Statement” field of the submission

- The written consent was be obtained
- The statements showing that the Institutional Review Board (IRB) approved use of oral consent was written in revised manuscript
- The oral consents was clearly documented in separate checklists

***Editor comment:*** We suggest you thoroughly copyedit your manuscript for language usage, spelling, and grammar.

*Author response:* We have edited the English grammar throughout the manuscripts

***Editor comment:*** Regarding the data availability statement

*Author response:* We have updated the data availability statement in which it is restricted

***Editor comment:*** Regarding to text overlap

*Author response:* The manuscript was paraphrased to minimize the texts overlap

***REVIEWER COMMENTS***

Reviewer 1

**Reviewer comments:** The conclusions must be drawn appropriately based on the data presented.

*Author response:* The conclusion was edited to relate with data presented

**Reviewer comments:** Regarding statistical analysis

*Author response:* The data was re-analyzed

**Reviewer comments:** Is the manuscript presented in an intelligible fashion and written in Standard English?

*Author response:* We have edited the English grammar throughout the manuscripts

**Reviewer comments:** The manuscript needs proof reading. There are awkward sentences that are difficult to understand that can be easily fixed at a proof reading stage. For example the first sentence in the abstract is not correct. Also in many places through the manuscript words that shouldn’t be capitalized are capitalized.

*Author response:* The manuscript was edited carefully and the first sentence in the abstract was corrected. Finally any capitalization was corrected

**Reviewer comments:** In many instances of importance to evaluate the manuscript rigor the provided text is missing critical information. For example the author say in the method section, it says a questionnaire was developed. But then the authors reference medical chart review. The connection between the different steps need to be further developed to allow the reader to understand what was actually done.

*Author response:* It was written as data was collected using questionnaire which was developed after reviewing different literature and checklist was prepared to verify the patient’s medical information in revised manuscript

**Reviewer comments:** Don’t capitalize the P in pharmaceutical

*Author response:* The capitalization was removed

**Reviewer comments:** Replace DTP by DRP

*Author response:* In abstract under result DTP was replaced by DRP

**Reviewer comments:** Re word A total of 331 DRPs were identified on average, 1.057 DRPs per

Patient to a total of 331 DRPs were identified, an average of 1.06 DRPs per patient.

*Author response:* It was written as per your comment

**Reviewer comments:** This sentence doesn’t make sense. Maybe something like the three-leading categories of DRPs were found to be unnecessary (not sure unnecessary what prescription of drug or consumption of drugs) with 92 cases (27.79%), non-adherence with 57 patients (17.22%) and high drug dosage with 56 cases (16.92%).

*Author response:* It was corrected as prescribed drugs

**Reviewer comments:** Is the term drug therapy problem the same as DRPs?

*Author response:* We have used them interchangeably

**Reviewer comments:** missing reference on ADR and The connection between these two sentences is not clear.Also two sentences can’t make a paragraph you need at least 5. I think the authors are trying to convey the significance of the problem and that if 5-20% patients may lose trust in the physicians treating them. But the authors never explain why that is a bad thing or make the actual connection.

*Author response:* The sentence regarding ADR was cited

- The two sentence was connected appropriately and many references was added to explain more about DRP
- We have discussed the reasons for high burden of ADR

**Reviewer comments:** What treatment that was poorly controlled? This thought is not complete

*Author response:* It was corrected in revised manuscript as hypertensive patient’s was poorly controlled

**Reviewer comments:** This time frame in of concern since the pandemic really started. Wouldn’t that impact the stress of physicians and health workers. Should be addressed in the limitaitons

*Author response:* The impact of COVID-19 on stress of physicians was written as limitation

**Reviewer comments:** You never explain ADR

*Author response:* ADR was assessed by using Naranjo algorithm of ADR probability scale. Accordingly, ADR Probability Scale was categorized by taking sum of 10 questions and grouped as definite, probable, possible or doubtful, if the total score is ≥9, 5-8, 1-4 and 0 respectively.It is mentioned as ”Regarding to the patients that were developed ADR, about 4(50%), 3(37.5%),1(12.5%) were definite, probable, possible ADR’’.

Thanks for your time and consideration,

Regards!
